# Fuzheng Huayu Capsule Attenuates Hepatic Fibrosis by Inhibiting Activation of Hepatic Stellate Cells

**DOI:** 10.1155/2020/3468791

**Published:** 2020-05-12

**Authors:** Mei Wu, Yang Zhou, Sheng-Lan Qin, Li-Jing Lin, Jian Ping, Zhang Tao, Jing Zhang, Lie-Ming Xu, Jian Wu

**Affiliations:** ^1^Institute of Liver Diseases, Shanghai University of Traditional Chinese Medicine, Shanghai 201203, China; ^2^Department of Liver Diseases, Shuguang Hospital Affiliated to Shanghai University of Traditional Chinese Medicine, Shanghai 201203, China; ^3^Department of Medical Microbiology, School of Basic Medical Sciences, Fudan University, Shanghai 200032, China; ^4^Shanghai Institute of Liver Diseases, Fudan University Shanghai Medical College, Shanghai 200032, China

## Abstract

**Aim:**

To investigate the mechanisms of Fuzheng Huayu (FZHY) Capsule in the treatment of hepatitis B (HBV)- associated fibrosis, HBV patients were divided into two groups, 50 cases were in the nucleotide analogues (NAs) group, while additional 50 cases were in the NAs + FZHY group.

**Methods:**

We assessed the curative effects of antifibrosis through liver function, FibroScan test, and liver biopsy and detected the ratio of lymphocyte subsets by flow cytometry. Peripheral blood lymphocyte and CD8^+^T, CD4^+^T, and natural killer cell subsets collected from patients were cocultured with LX-2 cells. Activation of LX-2 cells, production of the extracellular matrix, apoptosis, and proliferation of LX-2 cells were determined. Chronic liver injury models were established by ConA treatment.

**Results:**

It is evident that FZHY treatment significantly increased the percentage of NK cells, the rate of death, and apoptosis of LX-2 cells and decreased the FibroScan liver stiffness measurement value. The expressions of *α*-SMA and procollagen type I mRNA in LX-2 cells of the FZHY treatment group as downregulated when they were cocultured with lymphocytes compared to those from the NAs group. The proliferation of LX-2 cells in the FZHY treatment group was inhibited compared to that in the NAs group. In a mouse model of hepatic fibrosis, PBLs and IHLs from ConA exposure plus FZHY treatment inhibited the ability of JS-1 cells to express *α*-SMA.

**Conclusions:**

FZHY Capsule improved the disordered cellular immunity and postponed liver fibrosis possibly through inhibiting the interaction between lymphocyte and hepatic stellate cells.

## 1. Introduction

It is well known that various chronic liver diseases may be accompanied by hepatic fibrosis. Hepatic fibrosis (fibrosis of the liver) mainly results from hepatitis virus infection or alcoholism. It has been estimated that 350 million and 180 million individuals carry hepatitis B (HBV) and C (HCV) viruses in the world, respectively [1]. Moreover, hepatic fibrosis may develop into cirrhosis and eventually progress to death due to its complications.

In the initiation and progression of hepatic fibrosis, hepatic stellate cells (HSCs) play a critical role. HSCs proliferate massively, express various growth factors and receptors, and cause an imbalance between synthesis and degradation of collagen-based extracellular matrices (ECMs), leading to excessive deposit of ECM components in the liver, and finally result in the progression from hepatic fibrosis to cirrhosis [2, 3].

Patients with cirrhosis suffer from immune dysfunction. Recent studies suggested that T cells, in addition to participating in the inflammatory reaction of the liver, may activate HSCs directly without the involvement of cytokines [4, 5]. The influence of T cells on HSCs is supposed to be more complicated in viral hepatitis due to immune responses against the infection. In a coculture system, it was found that CD8^+^ and CD4^+^T cells of peripheral blood lymphocytes (PBLs) isolated from hepatitis B/hepatitis C patients accompanied with hepatic fibrosis are activated by adhering to HSCs and being phagocytosed [6]. It is speculated that cell-mediated adaptive immunity plays a crucial role in viral clearance. On the contrary, NK cells as part of the nonadaptive immunity may induce apoptosis of HSCs through a TRAIL- (tumor necrosis factor- (TNF-) related apoptosis-inducing ligand-) mediated mechanism [7, 8], which may be beneficial for the resolution of hepatic fibrosis. However, it is largely unknown how subsets of T cells participate in the immune response and interact with HCSs during the process of chronic viral infection.

“Fuzheng Huayu Capsule/tablet” is a patented Chinese herb medicine invented by the Institute of Liver Disease, Shanghai University of Traditional Chinese Medicine, after decades of laboratory and clinical investigations, and it effectively reverses hepatic fibrosis and cirrhosis as indicated in a number of clinical investigations [9, 10]. FZHYC also inhibited the angiogenesis of impaired liver and inhibited proliferation and function of HSCs, leading to suppression of hepatic fibrosis [9, 11].

Based on these clinical and laboratory investigations, we hypothesized that FZHYC may affect the function of PBLs by suppressing HSC activation and promoting HSC apoptosis in cirrhotic patients. In the present study, we investigate the effects of subsets of PBLs isolated from HBV-infected patients treated with nucleotide analogues (NAs) or NAs plus FZHYC treatment on HSCs. Our results demonstrated that PBLs from patients treated with NAs plus FZHYC were not as potent as those from patients receiving only NA treatment in terms of promoting HSC proliferation. Our animal experiments further confirmed the less promoting effects on HSCs by coculture with PBLs from mice receiving the combined treatment than NAs alone.

## 2. Materials and Methods

### 2.1. Patients

All enrolled cirrhotic subjects with chronic hepatitis B (CHB) were outpatients or inpatients aged from 18 to 80 who were cared in the Cirrhosis Department of Shuguang Hospital, Shanghai University of Traditional Chinese Medicine, from May 2012 to January 2017. Exclusive criteria include the following: (1) existence in other types of viral hepatitis or etiology and liver cancer; (2) severe diseases; (3) disabilities in the legal sense; (4) pregnant or lactating women, as well as those of childbearing potential not taking effective contraceptive measures; and (5) allergies to two or more drugs, foods, and pollen allergy or allergic to known components of this drug. Informed consent to all clinical investigations, in accordance with the principles outlined in the Declaration of Helsinki, was obtained from all enrolled patients. The study was approved by the Ethics Committee of Shuguang Hospital Affiliated to Shanghai University of Traditional Chinese Medicine (2013-253-22-01). Fifty patients in the NAs group were only treated with antiviral NAs for 12 ± 2 months, and additional 50 patients in the NAs + FZHY group were treated with NAs plus FZHYC for 12 ± 2 months. Twenty healthy individuals, volunteers from the Physical Examination Center of Shuguang Hospital, were enrolled in the control group. The demographic information of enrolled patients is described in Table 1. Mixed lymphocytes and lymphocyte subsets were isolated from 10 cases in the NAs group and NAs + FZHY group.

### 2.2. FZHY Capsule

FZHY Capsule (FZHYC) contains the herbal extraction mix that is composed of Dan Shen (Radix Salviae Miltiorrhizae), Chong Cao (*Paecilomyces hepiali* Chen & Dai), Tao Ren (Semen Persicae), Jiao Gu Lan (*Gynostemma pentaphyllum*), Song Hua Feng (*Pinus armandii* Franch.), and Wu Wei Zi (Fructus *Schisandra chinensis*). The composited capsules were manufactured by Shanghai Modern Pharmaceutical Company. The safety aspects such as adverse effects and NOAEL of FZHY Capsule in human subjects were evaluated previously [12]. The composition of 5 capsules (0.3 g per capsule) is equivalent to 24 g raw herbs. The dose was 15 capsules per day and they were divided into 3 times for oral administration. FZHYC is recommended to intake directly after a meal by CAIM (Chinese Association of Integrative Medicine). Some patients may experience discomfort in the abdominal area in a fasting condition. The antiviral NAs for HBV include Entecavir, Lamivudine, or Adefovir for oral administration with a daily dose of 0.5 mg, 100 mg, or 10 mg, respectively.

### 2.3. Reagents

Anti-human CD4-FITC, CD16-FITC, CD8a-PE, and CD56 (NCAM)-PE were from MultiSciences (Lianke) (Biotech Co. Ltd., Shanghai, CA). Phalloidin (FITC) (Alexis, Beijing, CA) and Alexa Fluor 488 Annexin V/Dead Cell Apoptosis Kit with Alexa Fluor 488 Annexin V and PI for Flow Cytometry and TRIzol (Invitrogen, Carlsbad) were used in the experiments. RevertAid™ First Strand cDNA Synthesis Kit (Fermentas) and quantitative chip (Quantibody Human Inflammation Array) (RayBiotech, Shanghai) were used in the RT-PCR analysis. Sword bean protein A (ConA) was purchased from Sigma Co., Ltd. ConA was dissolved in 10 mL NS as 2.5 mg/mL solution (12.5 mg/g, sterile operation, 0.45 mu *M* filter membrane filtration).

### 2.4. Laboratory Tests

The levels of serum albumin (Alb) and total bilirubin (TBiL), the activity of serum alanine aminotransferase (ALT), and aspartate aminotransferase (AST) were assayed with biochemical methods. The percentage (%) of CD3^+^CD4^+^T cells, CD3^+^CD8^+^T cells, and CD56^+^CD16^+^ (NK) cells in peripheral blood was determined by flow cytometry (Supplemental Figure 1).

### 2.5. Liver Cirrhosis Detection

All patients were examined with LSM by FibroScan before and after treatment. Liver biopsy and pathological observations were undertaken in twenty patients, including 12 cases in the NAs group and 8 cases in the NAs + FZHY group prior to and after the treatment (Supplemental Figure 2). Liver biopsies were performed using 16*G* biopsy needles. The biopsied specimens were fixed, paraffin-embedded, and stained with hematoxylin and eosin (H&E staining). A minimum of 1.5 cm of liver tissue with at least six portal tracts was required for diagnosis. Histological staging of the liver fibrosis (S0 to S4) was carried out according to Scheuer's criteria of hepatic fibrosis classification by three specialists [13].

### 2.6. Experimental Animals

C57BL/6NCrl Vr mice (male, 20∼22 g) were purchased from Beijing Vital River Laboratory Animal Technology Co. Ltd. The animals were maintained in the Experimental Animal Center, Shanghai University of Traditional Chinese Medicine, and kept in standard polypropylene cages. The approval for the animal experimental protocol was obtained from the Ethics Committee of Shuguang Hospital Affiliated to Shanghai University of Traditional Chinese Medicine (SZY201711008).

### 2.7. Isolation and Culture of Peripheral Blood Lymphocytes (PBLs)

Human blood at 3∼5 mL was collected with lithium heparin. PBLs were isolated by the density gradient centrifugation. Cells in the pellet were well resuspended in 1640 medium with 10% FBS and plated in plastic dishes. After standing for 2 h, cells in the supernatant were collected in new plastic dishes and pelleted cells were discarded. These cells in suspension were PBLs. The PBLs were suspended in 1640 medium with 10% FBS and plated in plastic dishes (100 mm in diameter) with a density of 1 × 10^6^ cells/mL. The cells were incubated at 5% CO_2_ and 95% humidified air at 37°C. The medium was replaced by 10% NBS-DMEM 24 h after seeding and renewed every 2-3 days.

### 2.8. Isolation of Intrahepatic Lymphocytes (IHLs)

The liver tissue of each mouse was fully minced, gently ground and filtrated with a piece of 100-mesh nylon, and then rinsed with HBSS solution. The further enrichment and seeding steps follow the procedure of PBL separation.

### 2.9. Isolation of Subsets of PBLs

PBL subsets were isolated from cirrhotic patients or healthy individuals by flow cytometry. Three to five milliliters of peripheral blood was collected with lithium heparin. After washing with PBS, anti-CD8^+^-PE, anti-CD4^+^-FITC, anti-CD56^+^-PE, and anti-CD16^+^-FITC antibodies were added to the cell suspension (Ebioscience). The subsets of PBLs were enriched by flow cytometry. Enriched cell fractions were counted with a counting plate and the viability of cells was about 95% as measured by the Trypan Blue Staining. The purity of CD8^+^T cells, CD4^+^T cells, or NK cells was 99.4%, 99.7%, or 96.8%, respectively (data not shown).

### 2.10. Culture of LX-2 Cells

LX-2 cells were suspended in DMEM medium with 10% FBS, plated in 6-well plates, 96-well plates or chambers, and incubated at 5% CO_2_ and 95% humidified air at 37°C. The medium was changed every 2-3 days. IHLs or subsets of lymphocytes were added to cultured LX-2 cell plates. The quantity ratio of LX-2 cells to lymphocytes was 1 : 1. The duration of the coculture was 24–48 h in the dark.

### 2.11. Cell Proliferation Assay (Staining with Alarma Blue)

LX-2 cells were incubated with 10% Alarma Blue medium for 6 hrs after cocultured with lymphocytes for 24 hrs. The fluorescent intensity was detected by an enzyme mark instrument at wavelengths of excitation (570 nm) and emission (600 nm)

### 2.12. Cell Activation Assay (FITC-Phalloidin)

Human LX-2 cells were incubated with FITC-Phalloidin for 2 hrs after cocultured with lymphocytes for 24 hrs. The myofilament was detected by confocal laser scanning microscopy at wavelengths of excitation (496 nm) and emission (516 nm).

### 2.13. Western Blot Analysis

20 *µ*g protein per lane was separated by 10–12% SDS-PAGE and electro-blotted onto a nitrocellulose membrane, which was probed with anti-*α*-SMA mouse antibodies (Kangcheng Biological Company, Shanghai, CA) and anti-GAPDH mouse antibodies (Kangcheng Biological Company, Shanghai, CA). IRDye 800CW donkey anti-mouse IgG and IRDye 680 conjugated goat anti-rabbit IgG (LiCor, CA, USA) was used to detect the bound mouse antibodies using an Odyssey test. GAPDH was used as a loading control.

### 2.14. Quantitative RT-PCR

Total RNA was extracted from LX-2 cells using TRIzol reagent (Invitrogen, Carlsbad, CA, USA). Cell complementary DNA (cDNA) was used to quantify mRNA levels of procollagen type I (COL.I) and GAPDH by q-PCR using the SYBRGreen Master Mix (Applied Biosystems, Foster, CA) using specific forward and reverse primers listed in Table 2. Human GAPDH mRNA levels served as an internal control to assess the overall cDNA content. The cycling parameters for the PCR program were as follows: initial denaturation step for 10 min at 95°C and then 40 cycles consisting of 10 s at 95°C, 5 s at 58°C, and 30 s at 70°C.

### 2.15. Detection of Apoptosis

Apoptosis of LX-2 cells was detected by (1) flow cytometry (FL1 FITC 530 nm, FL3 PI 575 nm) stained Annexin V and PI and analyzed using the FlowJo software; and (2) High Content Detection and analyzed using the ArrayScanVTI (700 series) software any staining of Hoechst33258.

### 2.16. Mouse Model of Hepatic Fibrosis

After one week of adaptability feeding, mice were divided into two groups: the control group (*n* = 5) and cirrhotic control group (*n* = 20). ConA was injected intraperitoneally at a dose of 12.5 mg/kg body weight once a week for 9 weeks in the cirrhotic group. Then, mice in the cirrhotic control group were randomly divided into the cirrhotic group (*n* = 10) and the FZHY treatment group (*n* = 10). Mice in the FZHY treatment group were given FZHY with a dose of 20 mL/kg body weight after 6 weeks of injection. Only ddH2O was fed to the mice in the normal control group and the cirrhotic control group with a volume of 20 mL/kg body weight. The administration was once a day for 4 weeks.

### 2.17. Pathological Observation of Mice

All mice were anesthetized with an intraperitoneal injection of 20% urethane and sacrificed. The liver specimen was embedded with paraffin. Sections at 4 *μ*m were prepared for H&E staining or Sirius Red Staining. Fibrosis scores were determined after the examination of three different areas of the tissue slide from each mouse. Hepatic fibrosis was semiquantitated according to Scheuer's criteria [13].

### 2.18. Statistical Analysis

The SPSS20.0 statistical software was used for data analysis and processing. The *χ*^2^ test was used for the counting data. The Ridit test was used for data from semiquantitative score analysis, the numerical data were expressed as mean ± SD, and the *t*-test was used for two sets of data. Variance analysis and *Q* tests were used for multiple comparisons of data between groups. *P* < 0.05 indicated that the differences were statistically significant.

## 3. Results

### 3.1. Alteration of Liver Functions and Liver Stiffness after the Treatment of FZHY Capsule

The activity of both ALT and AST was decreased after the treatment with NAs + FZHY. However, absolute changes in both ALT and AST activities were not statistically significant between NAs + FZHY and NAs groups. After treatment, serum Alb content was increased to some extent in both NAs + FZHY and NAs groups compared to the levels prior to the treatment. TBiL levels were mildly increased in both NAs and NAs + FZHY groups (Table 3).

**Figure 1:**
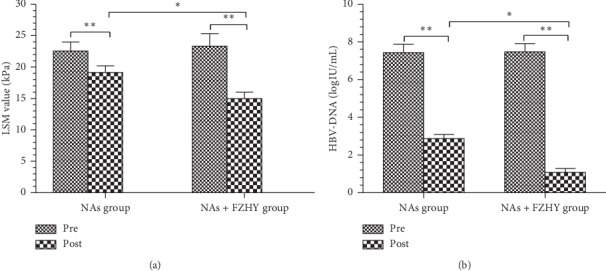
Fibrosis and HBV-DNA were decreased in the NAs + FZHY group after treatment. (a) LSM values were changed in both NAs and NAs + FZHY group before and after treatment. (b) HBV-DNA levels were significantly decreased in the FZHY group. ^*∗*^*P* < 0.05, ^*∗∗*^*P* < 0.01.

The FibroScan LSM value was 22.56 ± 10.14 kPa and 23.34 ± 13.91 kPa in NAs and NAs + FZHY groups before treatment, and it was significantly decreased to 19.14 ± 7.66 and 14.98 ± 7.28 kPa I (*P* < 0.01) in both groups after treatment (Figure 1(a)), suggesting that the addition of FZHY Capsule significantly improved fibrotic extent in HBV-infection patients while antiviral therapy was successful as indicated by the fact that HBV viral DNA load was dramatically decreased by 5 logs (Figure 1(b)).

Twenty patients were undertaken liver biopsy for pathological examination prior to and after the treatment, including 12 cases in the NAs group and 8 cases in the NAs + FZHY group. After treatment, the stage of liver fibrosis (S4) was significantly decreased in the NAs + FZHY group (Table 4).

### 3.2. The Effect of FZHY Treatment on Proportion of PBL Subsets in Patients

After treatment, the proportion of CD4^+^T cells was remarkably lower in the patients of both NAs and NAs + FZHY groups than that in healthy individuals. The difference in CD8^+^T proportion was not significant among the three groups. The fraction of NK cells, however, was much higher in the NAs + FZHY group than that in the NAs group.

There were 35 patients with a complete set of clinical data of cell-mediated immunity at the time points of pre- and posttreatment. There was no significant difference in the proportion of PBL subsets between pre- and posttreatment in the NAs group. The proportion of PBL subsets was increased at the posttreatment in the NAs + FZHY group in which CD4^+^T cell fraction was increased by 5.22 ± 9.96% compared to that of the pretreatment (Table 5).

### 3.3. Alteration of Proliferation and Function of LX-2 Cells Cocultured with PBLs

**Figure 2:**
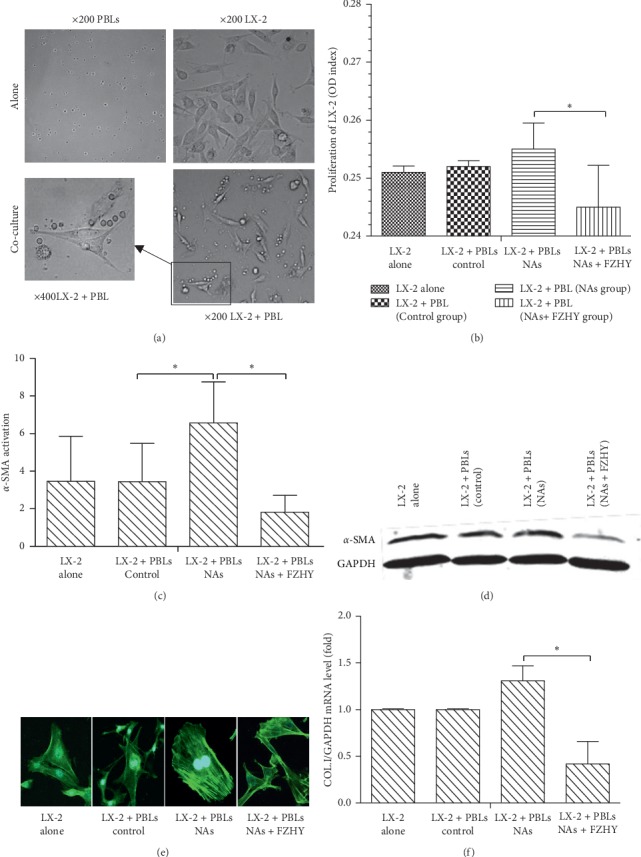
The proliferation ability and biology functions of LX-2 cells cocultured with PBLs were inhibited in the NAs + FZHY group compared with those in the NAs group. (a) The morphology of LX-2 cells was changed after cocultured with PBLs which were isolated from patients in the NAs + FZHY group. (b) Proliferation assay of LX-2 cells cocultured with PBLs which were isolated from patients in the normal group, NAs group, or NAs + FZHY group. (c) and (d) The protein expression levels of *α*-SMA in LX-2 cells were detected and analyzed by western blot analysis. (e) The relative mRNA expression level of collagen I in LX-2 cells was detected by qRT-PCR. (f) The myofilament stained FITC-Phalloidin was detected by confocal laser scanning. Microscope (green), ^*∗*^*P* < 0.05 LX-2+PBL (NA) versus LX-2+PBL (NA + FZHYC).

After cocultured with PBLs which were isolated from patients in NAs, NAs + FZHY, and control (healthy individuals) groups, LX-2 cells were morphologically characterized with extending pseudopodia, fusiform, or stellar. PBLs in suspension growth were seen as a small round shape, precipitating on the bottom of culture dishes due to gravity. The cells tended to get closer to LX-2 cells under light microscope (Figure 2(a)).

PBLs were isolated from three groups of patients (control, NAs, and NAs + FZHY). After cocultured with PBLs isolated from patients in the NAs + FZHY group, LX-2 cell proliferation was remarkably inhibited compared to those cultured with PBLs from the NAs group (Figure 2(b)). *α*-SMA expression of LX-2 cells was significantly increased when LX-2 cells were cocultured with PBLs from patients in the NAs group compared to the control group. The PBLs isolated from patients with FZHY treatment, however, appeared to have less action on *α*-SMA expression in LX-2 cells than those from the NAs group (Figures 2(c) and 2(d)). Meanwhile, myofilament expression in LX-2 cells also decreased significantly in the NAs + FZHY group (Figure 2(e)).

Although LX-2 cells are partially activating HSCs, the expression of procollagen I *α*1 mRNA of LX-2 cells was further increased during the coculture with PBLs in the NAs group compared to LX-2 cells without coculture. However, PBLs isolated from the patients in the NAs + FZHY group significantly inhibited procollagen I mRNA expression in LX-2 cells compared to those from the NAs group (Figure 2(f)).

### 3.4. Alteration in Proliferation and Function of LX-2 Cells Cocultured with Lymphocyte Subsets

**Figure 3:**
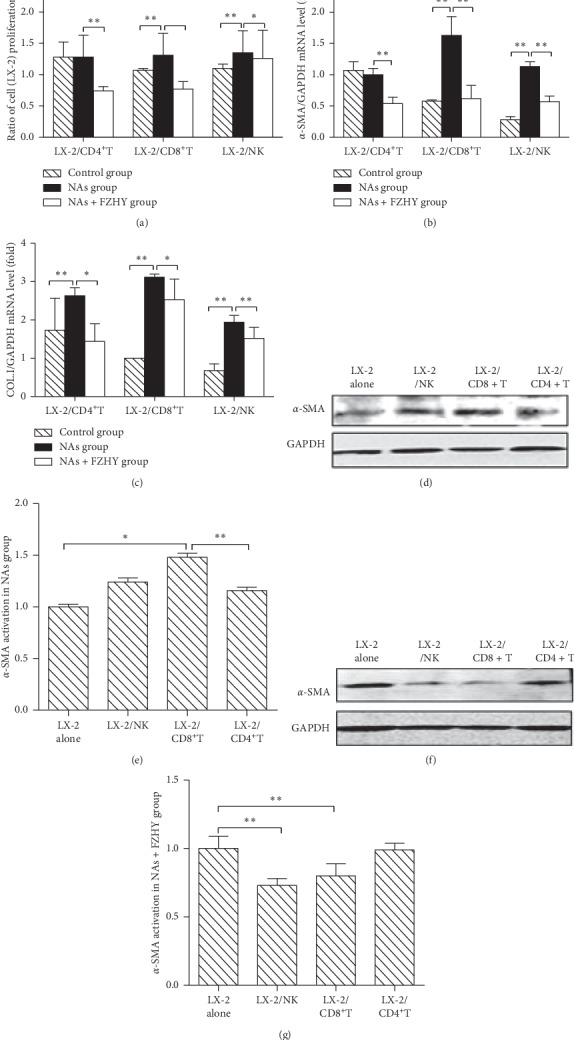
The expressions of *α*-SMA in LX-2 cells cocultured with PBLs subsets were decreased in the NAs + FZHY group compared with those in NAs group. (a) The proliferation of LX-2 cells cocultured with PBLs subsets which were isolated from patients in the normal group, NAs group, or NAs + FZHY group. (b) The relative mRNA expression level of *α*-SMA in LX-2 cells was detected by qRT-PCR. (c) The relative mRNA expression level of collagen I in LX-2 cells was detected by RT-PCR. (d) and (e) The protein expression level of *α*-SMA in LX-2 cells in the NAs group was detected and analyzed by western blot analysis. (f) and (g) The protein expression level of *α*-SMA in LX-2 cells in the NAs + FZHY group was detected and analyzed by western blot analysis. ^*∗*^*P* < 0.05, ^*∗∗*^*P* < 0.01.

Both CD8^+^T cells and NK cells, which were isolated from the NA-treated individuals, significantly promoted the proliferation of LX-2 cells compared to those from healthy individuals. In contrast, both CD4^+^T cells and CD8^+^T cells, which were isolated from the patients treated with FZHYC, inhibited LX-2 cell proliferation. However, NK cells did not stimulate the proliferation of LX-2 cells to a significant extent. LX-2 cell proliferation stimulated by NK cells from the NAs + FZHY group was less profound than that from the NAs group (Figure 3(a)).

CD8^+^T and NK cells from patients in the NAs group significantly stimulated *α*-SMA mRNA expression in LX-2 cells compared to those from the control subjects. In contrast, CD4^+^T, CD8^+^T, and NK cells significantly inhibited *α*-SMA mRNA expression in LX-2 cells from the NAs + FZHY group compared to those from the NAs group (Figure 3(b)).

Each PBL subset from the NAs group obviously stimulated procollagen I mRNA expression of LX-2 cells compared to those from the control subjects. However, CD4^+^T, CD8^+^T, or NK cells from the NAs + FZHY group inhibited procollagen I mRNA expression of LX-2 cells compared to those from the NAs group (Figure 3(c)).

*α*-SMA protein expression of LX-2 cells was significantly increased when they were cocultured with CD8^+^T cells from the NAs group compared to those cultured alone. However, NK or CD4^+^T cells from the NAs group did not significantly increase the *α*-SMA protein expression of LX-2 cells (Figures 3(d) and 3(e)). CD8^+^T and NK cells isolated from the patients in the NAs + FZHY group significantly inhibited the *α*-SMA protein expression of LX-2 cells compared to those in LX-2 cells cultured alone (Figures 3(f) and 3(g)).

### 3.5. Alteration in LX-2 Cell Apoptosis when Cocultured with PBL Subsets

**Figure 4:**
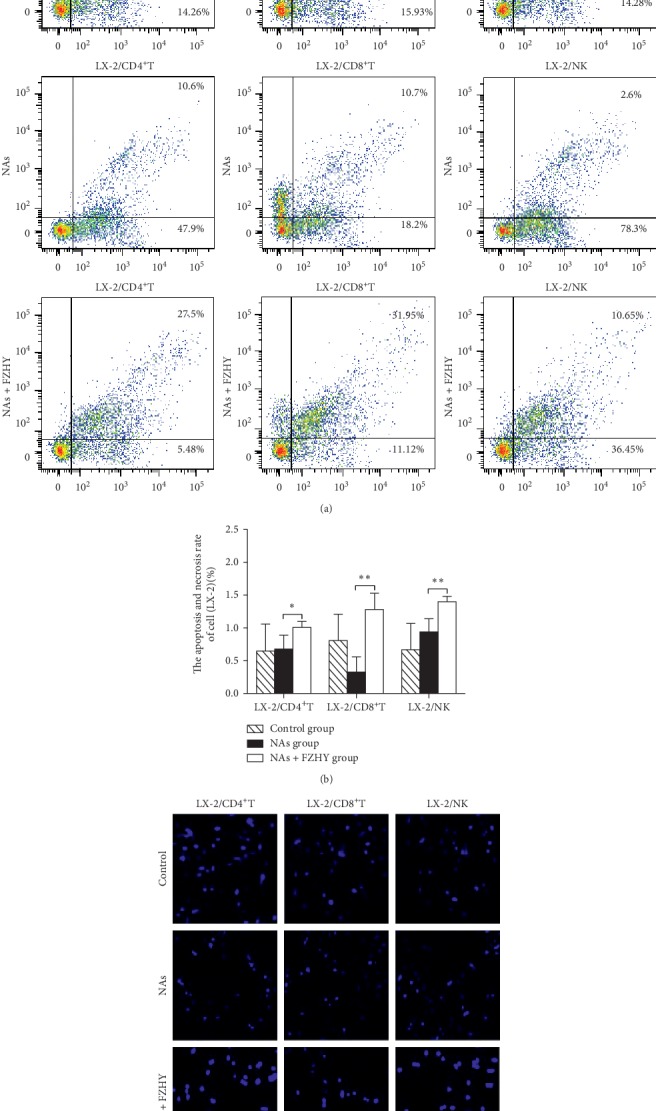
The apoptosis of LX-2 cells cocultured with PBL subsets was increased in the NAs + FZHY group compared with that in the NAs group. (a) The proportion of apoptosis and necrosis in LX-2 cells cocultured with PBL subsets was detected by flow cytometry. (b) The statistical analysis for the apoptosis and necrosis in LX-2 cells in all groups according to the results of flow cytometry. (c) The cell fluorescent imaging for the cell nucleus of LX-2 cells was stained by Hoechst 33258. Magnification, ×200. ^*∗*^*P* < 0.05, ^*∗∗*^*P* < 0.01.

Apoptosis and necrosis of LX-2 cells were detected by flow cytometry (Figure 4(a)). Decreased apoptosis and necrosis in LX-2 cells were observed after cocultured with CD8^+^T cells; however, both were increased after cocultured with NK cells from the NAs group (Figures 4(a) and 4(b)). Moreover, apoptosis and necrosis of LX-2 cells were increased after cocultured with CD4^+^T, CD8^+^T, or NK cells from the NAs + FZHY group (Figures 4(a) and 4(b)).

**Figure 5:**
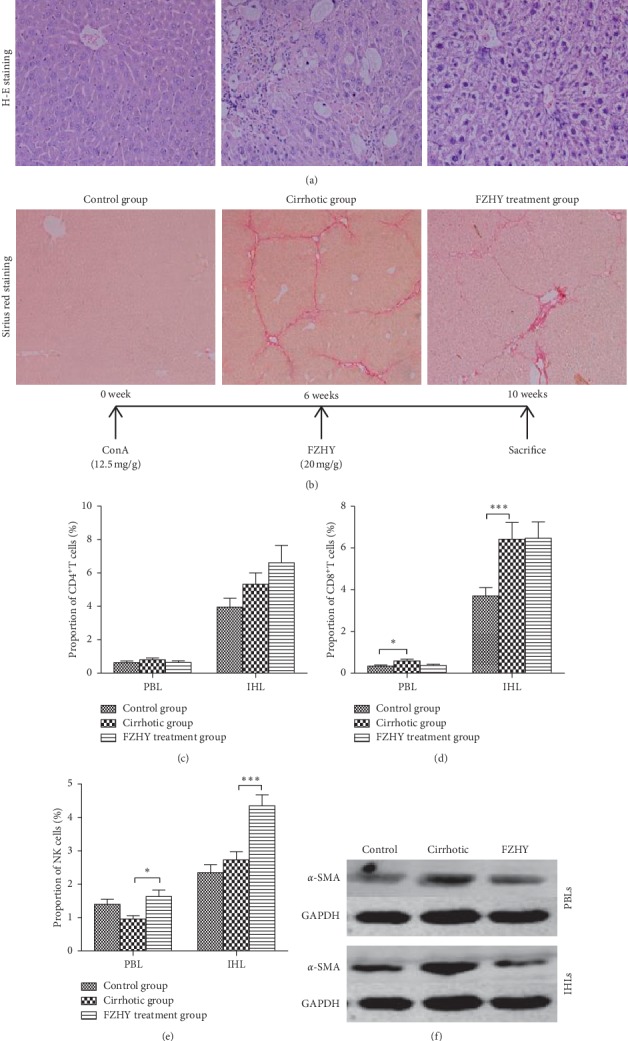
FZHY could suppress the hepatic fibrosis in vivo. (a) The paraffin sections of the mouse livers were stained by H&E staining (×200). (b) The paraffin sections of the mouse livers were stained by Sirius Red Staining (×100). (c–e) The proportion of mouse PBL or IKL subsets in each group. (f) The protein expression levels of *α*-SMA in JS-1 cells which were cocultured with PBLs or IHLs that were isolated from the cirrhotic mouse in different groups.

Hoechst 33258 staining showed that both CD8^+^T and CD4^+^T cells significantly decreased the fluorescent intensity of LX-2 cell nuclei (apoptotic cells) in the NAs group compared to those from the control subjects. On the contrary, CD4^+^T, CD8^+^T, or NK cells significantly increased the apoptosis of LX-2 cells in the NAs + FZHY group compared to those in the NAs group (Figure 5(c)).

### 3.6. Effect of FZHY Extract on Hepatic Fibrosis in Mice

The mouse cirrhotic model was successfully established after ConA injections, and severe inflammation with ballooning degeneration of hepatocytes and obvious pseudo-lobules surrounded by fibrous septa were observed in the liver section (Figures 5(a) and 5(b) and Table 6). The degree of ballooning degeneration, hepatocellular death, and infiltration of inflammatory cells was remarkably decreased in the FZHY treatment group compared to that in the cirrhotic mice. The fibrotic deposition was significantly improved in the FZHY treatment group compared to that in the cirrhotic mice.

Are the effects of PBLs the same as intrahepatic lymphocytes (IHLs) during the treatment of cirrhotic patients with FZHYC? In other words, could the alteration of PBL numbers and functions reflect that of IHLs during the treatment of cirrhosis with FZHYC? To answer these questions, we isolated PBLs and IHLs from all mice and then cocultured them with mouse hepatic stellate JS-1 cells, respectively. The proportions of PBLs were altered in the way almost similar to IHLs in the cirrhotic mice. The proportions of CD4^+^T and CD8^+^T cells in PBLs and IHLs were increased in the cirrhotic group. In the FZHY treatment group, the proportion of NK, CD4^+^T, and CD8^+^T cells was significantly increased in PBLs and IHLs compared with the cirrhotic group. The trend of changes was similar in PBLs and IHLs of cirrhotic mice treated with FZHY (Figures 5(c)–5(e)).

*α*-SMA expression of mouse JS-1 cells cocultured with PBLs or IHLs from the cirrhotic mice was increased compared to that from the controls (Figure 5(f)). However, compared with the cirrhotic group, *α*-SMA expression of JS-1 cells cocultured with PBLs or IHLs in the FZHY treatment group was reduced (Figure 5(f)).

## 4. Discussion

Fuzheng Huayu Capsule (FZHYC) is composed of 6 Chinese herb medicines. *Salvia miltiorrhiza* could suppress cellular immunity and humoral immunity, reduce the infiltration of immune cells in inflammation of the area and regulate the secretion of IL-10 [14]. Fermentative cordycepic fungal powder could regulate cellular immunity by enhancing the function of T cells and activity of NK cells or inhibiting the secretion of IL-1 and IL-2 [15, 16]. Pathological observation showed the reversal of hepatic fibrosis after the treatment of chronic hepatitis B with FZHYC [9]. By promoting the activity of collagenase, it may accelerate the degradation of ECMs in the liver and promote the regression of hepatic fibrosis [17]. In clinical observation, it was found that Fuzheng Huayu Capsule not only exhibited an antifibrotic effect but also corrected abnormalities in the cellular immunity of cirrhotic patients [9]. These results suggested that FZHYC improves the function of cell-mediated immunity. A Phase II, randomized, placebo-controlled, double-blind, multicenter study was successfully completed to assess the antifibrotic activity of Fuzheng Huayu tablet in patients with chronic hepatitis C plus hepatic fibrosis in the United States in 2013. However, how FZHYC affects hepatic fibrogenesis through immune regulatory mechanisms is unclear.

In the present study, we found that the total number and proportions of isolated PBLs and their subsets were significantly different in HBV-infected patients who were treated with NAs alone or NAs plus FZHYC. Then, the PBLs were cocultured with LX-2 cells, and we found that the morphology and function of LX-2 cells were dramatically changed in the direction of activation.

Given that the regulation of lymphocytes on the activation of HSCs depends on the function of lymphocyte subsets and that CD4^+^T cells have been implicated in the pathogenesis of hepatic fibrosis [18], we observed that the number of CD4^+^T cells was lower in the patients of the NAs group and NAs + FZHY group than that from the control subjects. In contrast, the number of NK cells was obviously increased in the patients of the NAs + FZHY group than that from the NAs group. NK cells are a key component of the innate immune system and possess antifibrotic activity through direct HSC killing [19–21]. Therefore, our results indicated that the increase of the CD4^+^T and NK cells and/or decrease of the CD8^+^T cells could be a potential mechanism through which FZHYZ modulates fibrosis in HBV-infected patients.

Fibroscan LSM value appears to be a reliable parameter for the noninvasive diagnosis of advanced fibrosis and cirrhosis and has become an alternative approach over an invasive approach of liver biopsy [22–25]. This study showed that the decrease in the LSM value was significantly greater in the NAs + FZHY group than in the NAs group after the completion of NAs plus FZHY treatment, further confirming the antifibrotic effects of FZHYC treatment. Our data also demonstrated that there is a strong negative correlation between NK cells and LSM value, as well as a moderate negative correlation between the ratio of NK/CD8^+^T and LSM value (data not shown). In addition, CD4^+^T cell count was significantly increased after the completion of the treatment compared to that of the pretreatment in the NAs + FZHY group, and the numbers of NK cells were significantly increased in the patients at the posttreatment in the NAs + FZHY group, indicating that the underlying mechanisms of FZHYC effects on hepatic fibrosis are associated with immunologic modulation.

*α*-SMA is a marker for active HSCs [26], and synthesis and secretion of collagen I by HSCs are key events during hepatic fibrogenesis [27]. *α*-SMA expressions of LX-2 cells cocultured with PBLs in the NAs group were increased compared to those cultured alone whereas the expressions of *α*-SMA, procollagen I, and LX-2 cell proliferation when cocultured with PBLs from the NAs + FZHY group were decreased compared to those from the NAs group. Of note, both CD4^+^ and CD8^+^T cells, which were isolated from the patients treated with FZHYC, significantly inhibited LX-2 cell proliferation, suppressed *α*-SMA and procollagen I expression, and promoted apoptosis and necrosis of LX-2 cells during coculture. The activation of LX-2 cells was inhibited whereas apoptosis and necrosis of LX-2 cells were promoted during coculture of LX-2 cells with NK cells from the NAs + FZHY group compared to those from the NAs group. Therefore, it is convincing that FZHYC suppressed the activation of HSCs partially by altering T cells in both number and function.

There were similar changes in PBLs subsets and IHLs subsets in a mouse model of liver cirrhosis. The proportion of CD8^+^T cells was significantly increased in cirrhotic mice in both PBLs and IHLs. Both PBLs and IHLs, which were isolated from the same cirrhotic mouse, significantly induced *α*-SMA expression during these lymphocytes cocultured with mouse JS-1 HSC cells, respectively. It is indicated that the trend of influence of PBLs and IHLs on JS-1 cell activation was similar in cirrhotic mice. These results suggested that PBLs could replace IHLs under certain conditions to investigate the relationship between T lymphocytes and HSCs.

The effects of FZHY extract on hepatic fibrosis were clearly demonstrated in cirrhotic mice in the present study. The liver pathology, especially in liver injury and fibrosis, was significantly improved in the FZHY treatment group compared with cirrhotic mice. The proportion of NK cells, in PBLs or IHLs isolated from mice of the FZHY treatment group, was significantly greater than that of the cirrhotic group. However, the proportion of CD4^+^ or CD8^+^T cells was different in the FZHY treatment group from the cirrhotic group. Coculture with PBLs or IHLs isolated from the same mouse in the FZHY treatment group resulted in reduced expression of *α*-SMA in mouse HSCs compared to that of the cirrhotic group. The results of the mouse experiments are in great accordance with clinical investigation and experiments.

## 5. Conclusions

In conclusion, FZHY capsule reversed the imbalance of cell-mediated immunity in patients who suffered from chronic hepatitis B with fibrosis/cirrhosis. FZHYC suppressed proliferation and activation of HSCs indirectly and promoted apoptosis and necrosis of HSCs by modulating the proportion and function of T lymphocyte subsets, thus contributing to block fibrotic progression. In addition, PBL has great potential to replace IHLs and could be useful for investigating mechanisms of therapeutics on antifibrosis.

## Figures and Tables

**Table 1:** Clinical characteristics.

Characteristics	Control group	NAs group	NAs + FZHY group
*n*	20	50	50
Sex (male/female)	12/8	28/22	30/20
Age (year)	60.30 ± 10.04	55.24 ± 10.13	56.69 ± 10.53
Partial response	—	7	2
Complete response	—	43	48

**Table 2:** Oligonucleotide sequences of primers utilized for qRT-PCR.

Target	Forward primer (5′-3′)	Reverse primer (5′-3′)
COL.I	CAAGAGGAACACATATGGAG	CCCAGAACATCACATATCAC
*α*-SMA	CCGACCGAATGCAGAAGGA	ACAGAGTATTTGCGCTCCGGA
GAPDH	CAAGTTCAACGGCACAGTCAAGG	ACATACTCAGCACCAGCATCACC

RT-PCR, reverse transcriptase-polymerase chain reaction; *α*-SMA, smooth muscle *α*-actin; COL.I, procollagen type I.

**Table 3:** Changes in ALT, AST, Alb, and TBiL in both groups before and after treatment.

	NAs group				NAs + FZHY group			
	*n*	Pre	Post	Post-Pre	n	Pre	Post	Post-Pre
ALT (IU/L)	50	54.04 ± 24.73	39.40 ± 19.02^▲^	−11.14 ± 25.72	50	49.30 ± 25.29	28.50 ± 9.90^*∗*^^△^	−21.89 ± 25.38
AST (IU/L)	50	59.79 ± 24.56	41.04 ± 29.93^▲^	−15.05 ± 36.42	50	53.77 ± 24.52	29.71 ± 13.60^*∗∗*^^△^	−23.55 ± 23.10
Alb (g/L)	50	35.38 ± 7.82	36.94 ± 8.09	2.92 ± 16.76	50	36.06 ± 7.96	35.91 ± 9.04	−1.66 ± 15.76
TBiL (mmol/L)	50	25.03 ± 12.85	26.20 ± 16.12	2.64 ± 16.18	50	25.97 ± 12.39	26.03 ± 14.89	−2.11 ± 12.83

^*∗*^ *P* < 0.05, ^▲^*P* < 0.01 versus the Pre of NAs group; ^Δ^*P* < 0.01versus the Pre of NAs + FZHY group. Alb, serum albumin; TBiL, serum total bilirubin; ALT, serum alanine aminotransferase; AST, serum aspartate aminotransferase.

**Table 4:** Liver fibrosis staging of two groups before and after treatment (according to the criteria of Scheuer's classification).

	NAs group		NAs + FZHY group	
*n*	Pre	Post	Pre	Post
S1	0 (0%)	0 (0%)	0 (0%)	0 (0%)
S2	2 (25%)	0 (0%)	2 (16.7%)	2 (16.7%)
S3	4 (50%)	3 (37.5%)	7 (58.3%)	8 (66.7%)
S4	2 (25%)	5 (62.5%)	3 (25%)	2 (16.7%)^*∗*^
Total	8	8	12	12

^*∗*^ *P* < 0.05 versus the Post S4 of NAs group; S, stage of liver fibrosis (S0–S4).

**Table 5:** Changes in the proportion of PBL subsets in patients before and after treatment (%).

	NAs group				NAs + FZHY group			
	*n*	Pre	Post	Post-Pre	n	Pre	Post	Post-Pre
CD4^+^T cells	35	35.72 ± 10.10	35.40 ± 10.68	−0.32 ± 11.98	35	32.16 ± 8.19	37.39 ± 9.17^*∗*^	5.22 ± 9.96^△^
CD8^+^T cells	35	31.55 ± 9.38	32.72 ± 10.52	1.17 ± 12.71	35	30.18 ± 5.58	28.89 ± 9.55	−1.28 ± 10.97
NK cells	35	16.43 ± 7.13	16.70 ± 9.25	0.31 ± 5.99	35	17.64 ± 7.15	21.14 ± 8.97^△△^	3.19 ± 5.12

^*∗*^ *P* < 0.05 versus Pre CD4^+^T cells of NAs + FZHY group; ^Δ^*P* < 0.05 versus Post-Pre CD4^+^T cells of NAs group; ^ΔΔ^*P* < 0.05 versus Pre NK cells of NAs + FZHY group. PBL, peripheral blood lymphocyte.

**Table 6:** Liver fibrosis staging of three groups after treatment (according to the criteria of Scheuer's criteria).

*n*	Control group	Cirrhotic group	FZHY treatment group
S0	5 (100%)	0 (0%)	0 (0%)
S1	0 (0%)	0 (0%)	1 (10%)
S2	0 (0%)	1 (10%)	5 (50%)
S3	0 (0%)	5 (50%)	3 (30%)
S4	0 (0%)	4 (40%)	1 (10%)^*∗*^
Total	5	10	10

^*∗*^ *P* < 0.05 versus S4 of cirrhotic group; S, stage of liver fibrosis (S0–S4).

## Data Availability

The data used to support the findings of this study are available from the corresponding author upon request.

## Abbreviations

Alb: Serum albumin
TBiL: Serum total bilirubin
ALT: Serum alanine aminotransferase
AST: Aspartate aminotransferase
CHB: Chronic hepatitis B
COL.I: Collagen type I
Con A: Concanavalin A
ECM: Extracellular matrix
FZHYC: Fuzheng Huayu Capsule
FZHYE: Fuzheng Huayu Extract
GAPDH: Glyceraldehydes phosphate dehydrogenase
HBV: Hepatitis B virus
HSC: Hepatic stellate cell
IHL: Intrahepatic lymphocyte
LSM: Liver stiffness measurement
NAs: Nucleoside analogues
NK cell: Nature killer cell
PBMC: Peripheral blood mononuclear cell
PBL: Peripheral blood lymphocyte
RT-PCR: Reverse transcriptase-polymerase chain reaction
SMA: Smooth muscle *α*-actin.

## Supplementary Materials

Supplementary Materials Supplemental Figure 1: purity identification of separated PBL subsets (CD4^+^T cells, CD8^+^T cells, and NK cells). Supplemental Figure 2: FZHY could suppress the hepatic fibrosis in patients.
