# Supplementary material for: Fuzheng Huayu Capsule Attenuates Hepatic Fibrosis by Inhibiting Activation of Hepatic Stellate Cells
Source: Evid Based Complement Alternat Med. 2020 May 12;2020:3468791. doi: 10.1155/2020/3468791 (PMC7243025; doi:10.1155/2020/3468791)
Supplement: Supplementary Materials — Supplemental Figure 1: purity identification of separated PBL subsets (CD4+T cells, CD8+T cells, and NK cells). Supplemental Figure 2: FZHY could suppress the hepatic fibrosis in patients. [file 3468791.f1.docx]

**Supplementary materials**

**
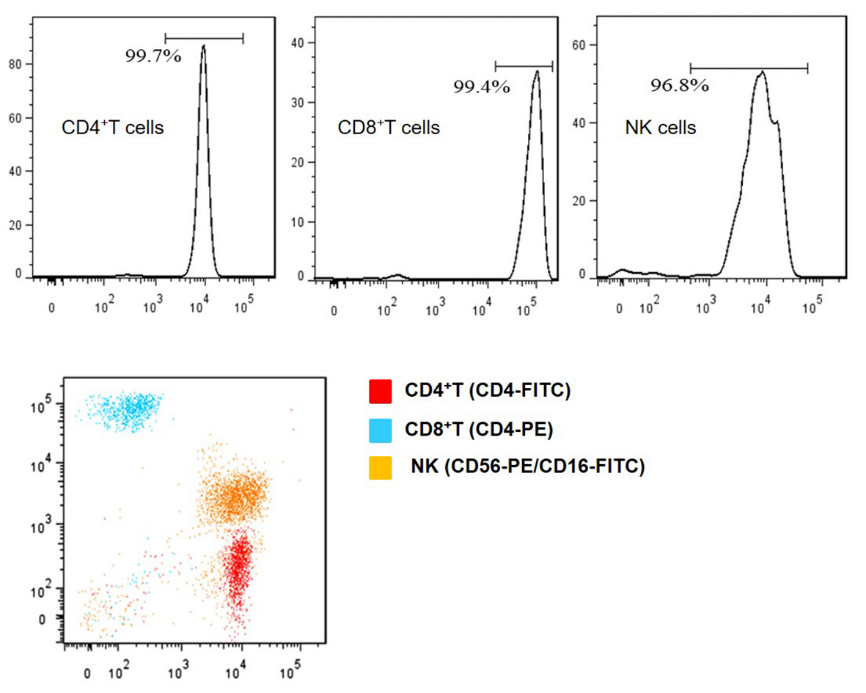
**

**Supplemental Figure 1 Purity identification of separated PBL subsets (CD4+ T cells，CD8+ T cells and NK cells).** PBLs were incubated with anti-CD8+-PE, anti-CD4+-FITC, NK (anti-CD16+-FITC/anti-CD56+-PE) antibodies respectively for 24 h. The PBL subsets were sorted by flow cytometry. CD8+ T cells were red round cells，CD4+ T cells were green round cells and NK cells were orange round cells.

**
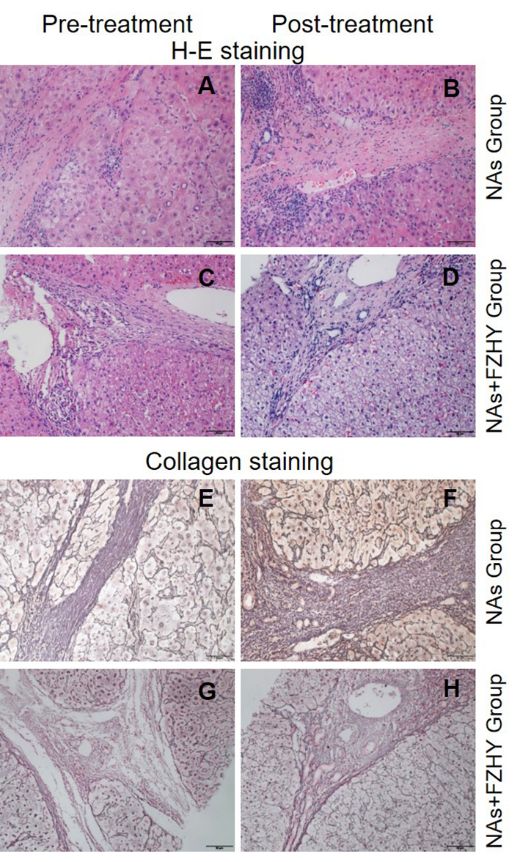
**

**Supplemental Figure 2 FZHY could suppress the hepatic fibrosis in Patients.** (A) The paraffin sections of the patient livers were stained by H-E staining before treatment in NAs group (×200). (B) The paraffin sections of the patient livers were stained by H-E staining after treatment in NAs group (×200). (C) The paraffin sections of the patient livers were stained by H-E staining before treatment in NAs+FZHY group (×200). (D) The paraffin sections of the patient livers were stained by H-E staining after treatment in NAs+FZHY group (×200). (E) The paraffin sections of the patient livers were stained by collagen staining before treatment in NAs group (×200). (F) The paraffin sections of the patient livers were stained by collagen staining after treatment in NAs group (×200). (G) The paraffin sections of the patient livers were stained by collagen staining before treatment in NAs+FZHY group (×200). (D) The paraffin sections of the patient livers were stained by collagen staining after treatment in NAs+FZHY group (×200).
